# Predictors of loss to follow-up in art experienced patients in Nigeria: a 13 year review (2004–2017)

**DOI:** 10.1186/s12981-019-0241-3

**Published:** 2019-10-08

**Authors:** Ahmad Aliyu, Babatunde Adelekan, Nifarta Andrew, Eunice Ekong, Stephen Dapiap, Fati Murtala-Ibrahim, Iboro Nta, Nicaise Ndembi, Charles Mensah, Patrick Dakum

**Affiliations:** 1 Institute of Human Virology Nigeria (IHVN), Abuja, Nigeria; 2 Academy for Health Development, Ife, Nigeria

**Keywords:** Loss to follow-up, ART, Nigeria

## Abstract

**Background:**

Expanded access to antiretroviral therapy (ART) leads to improved HIV/AIDS treatment outcomes in Nigeria, however, increasing rates of loss to follow-up among those on ART is threatening optimal standard achievement. Therefore, this retrospective cross-sectional study is aimed at identifying correlates and predictors of loss to follow-up in patients commencing ART in a large HIV program in Nigeria.

**Methods:**

Records of all patients from 432 US CDC Presidents Emergency Plan for AIDS Relief (PEPFAR) supported facilities across 10 States and FCT who started ART from 2004 to 2017 were used for this study. Bivariate and multivariate analysis of the demographic and clinical parameters of all patients was conducted using STATA version 14 to determine correlates and predictors of loss to follow-up.

**Results:**

Within the review period, 245,257 patients were ever enrolled on anti-retroviral therapy. 150,191 (61.2%) remained on treatment, 10,960 (4.5%) were transferred out to other facilities, 6926 (2.8%) died, 2139 (0.9%) self-terminated treatment and 75,041 (30.6%) had a loss to follow-up event captured. Males (OR: 1.16), Non-pregnant female (OR: 4.55), Patients on ≥ 3-monthly ARV refills (OR: 1.32), Patients with un-suppressed viral loads on ART (OR: 4.52), patients on adult 2nd line regimen (OR: 1.23) or pediatric on 1st line regimen (OR: 1.70) were significantly more likely to be lost to follow-up.

**Conclusion:**

Despite increasing access to anti-retroviral therapy, loss to follow-up is still a challenge in the HIV program in Nigeria. Differentiated care approaches that will focus on males, non-pregnant females and paediatrics is encouraged. Reducing months of Anti-retroviral drug refill to less than 3 months is advocated for increased patient adherence.

## Background

Loss-to-follow up (LTFU)—a situation where people living with HIV (PLHIV) receiving combination antiretroviral therapy (cART) become unaccounted for within a specified period, remains a frequent clinical and epidemiological challenge for HIV programs [1, 2]. LTFU is associated with poorer consequences [2–4] because of loss of PLHIV across the care continuum and complicates global evidence of the rapid ART scale up era [5, 6]. As at the end of 2017, an estimated 21.7 million [19.1–22.6 million] people globally were accessing treatment [7], and in Africa’s hard hit eastern and southern regions covering 10.3 million people, a 24% [22–26%] to 54% [50–58%] rise. As at 2016, about 30% ~ 960,000 (608,000–1,344,000) of Nigeria’s 3,200,000 (2,300,000–4,300,000) PLHIV were on cART [8].

Notwithstanding regional, age, gender and population-risk disparities, epidemic-control gains of the cART era are apparent, with worldwide AIDS-related deaths down by 34% from 2010 to 2017 and stagnation in incident HIV infections (all ages), which declined from a peak of 3.4 million [2.6–4.4 million] in 1996 to 1.8 million [1.4–2.4 million] in 2017 [7]. Since 2010, new HIV infections and AIDS-related deaths in Nigeria, have slid by 21% and 6% respectively [8], with Nigeria in 2016, aiming to maximize gains by adopting the treat-all approach that mandates prompt and universal cART for all PLHIVs [9, 10].

Multiple studies abound on the magnitude of LTFU and its drivers in sub-Saharan Africa (SSA) with patient-related factors including gender, education, age, ART initiation, ART status, CD4 level, duration of diagnosis identified as major factors [11–14]. Results vary based on approach, definition, and period when the LTFU cohort were in care (pre-ART) or on ART. A systematic review of 39 LTFU cohorts and 22,6307 patients in SSA found attrition to be 22.6% at 12 months and 23% to 30% at 24 months [2], while another review of 180,718 patients across six regions reported a LTFU rate of 19.9% [3]. An analysis of 4206 patients initiated on ART in a large HIV program in Nigeria described that 24.8% of them were LTFU after 10 years [15] while a nationally representative study of patients on ART stated a LTFU prevalence of 12.3% per 100 person years [16].

By utilizing long-term routine program data to ascertain how patient, site and related factors impact LTFU, this study contributes to discourse of Nigeria’s LTFU issue in the context of HIV program improvement. Such knowledge has potential to inform program effectiveness and efficiency [1, 5, 8] as well as mitigate the consequences of LTFU.

## Methodology

### Study population and settings

Data of all PLHIV adults, pregnant women, pediatrics and adolescents who were enrolled within the Institute of Human Virology Nigeria (IHVN) network into care and received ART from 2004 to 2017 from 432 PEPFAR/CDC supported public health facilities (Tertiary, Secondary, Primary) and private health facilities across 10 Nigerian states (see Table 1) and Federal Capital Territory (FCT) were used for this study. The IHVN is an NGO that provides quality HIV care through technical assistance and support to Nigerian states.

**Table 1:** Socio-demographic factors and its association with LTFU and active status

Maternal factors	LTFU status		Total	p (< 0.05)
	No	Yes		
Sex				
Female	105,630 (67.69)	50,444 (32.31)	156,134 (69.32)	< *0.001*
Male	44,501 (64.40)	24,597 (35.60)	69,098 (30.68)	
State				
Benue	29,724 (66.28)	15,122 (33.72)	44,846 (19.91)	< *0.001*
Delta	11,633 (63.90)	6572 (36.10)	18,205 (8.08)	
Ekiti	2812 (74.91)	942 (25.09)	3754 (1.67)	
FCT	39,301 (65.74)	20,482 (34.26)	59,783 (26.64)	
Kaduna	2252 (54.03)	1916 (45.97)	4168 (1.85)	
Kano	10,960 (71.98)	4266 (28.02)	15,226 (6.76)	
Katsina	6430 (63.60)	3680 (36.40)	10,110 (4.49)	
Nassarawa	28,196 (65.61)	14,777 (34.39)	42,972 (19.08)	
Ogun	7181 (69.56)	3143 (30.44)	10,324 (4.58)	
Ondo	8407 (73.03)	3105 (26.97)	11,512 (5.11)	
Osun	3296 (76.08)	1036 (23.92)	4332 (1.92)	
Age at ART start (years)				
1–9			8762 (3.89)	
10–14			2560 (1.14)	
15–19			4121 (1.83)	
20–24			19,608 (8.71)	
25–49			169,341 (75.19)	
50–64			18,716 (8.31)	
65+			2124 (0.94)	
Age at endline (years)				
1–9	4240 (73.14)	1557 (26.86)	5797 (2.57)	< *0.001*
10–14	2354 (68.23)	1096 (31.77)	3450 (1.53)	
15–19	2326 (65.99)	1199 (34.01)	3525 (1.57)	
20–24	7599 (68.85)	3438 (31.15)	11,037 (4.90)	
25–49	110,590 (66.70)	55,207 (33.30)	165,797 (73.61)	
50–64	20,332 (65.49)	10,716 (34.51)	31,048 (13.78)	
65+	2750 (60.07)	1828 (39.93)	4578 (2.03)	
Female status				
Breastfeeding	1523 (84.44)	199 (11.56)	1722 (1.10)	< *0.001*
Pregnant	100,142 (67.08)	49,137 (32.92)	149.279 (95.61)	
Not pregnant	4025 (78.41)	1108 (21.59)	5133 (3.29)	
Region				
North^a^	116,862 (65.98)	60,243 (34.02)	177,105 (78.63)	< *0.001*
South^b^	33,329 (62.25)	14,798 (30.75)	48,127 (21.37)	
Northern region				
North central^c^	97,220 (65.87)	50,381 (34.13)	147,601 (83.34)	*0.019*
North west^d^	19,642 (65.98)	9862 (33.43)	29,504 (16.66)	

p = p value which is in italics if significant at < 0.05

^a^North also referred as Northern region: Benue, FCT, Kaduna, Kano, Katsina and Nassarawa

^b^South also referred as Southern region: Delta, Ondo, Ogun, Osun and Ekiti

^c^North central: Benue, FCT and Nassarawa

^d^North west: Kano, Kaduna and Katsina

### Study design and implementation

The study is a retrospective cross-sectional study of clinical data of patients ever enrolled on ART.

### Data collection/analysis

All patients reported on the Retention and Audit Determination Tool (RADET) from January 2004 to June 2017 were extracted into an excel template. Demographic and clinical program parameters of all patients were described using frequencies and percentages. Bi-variate and multivariate analysis using Chi square and logistic regression was conducted for patients that were active or LTFU. All p-values reported were two-sided. Analyses were conducted in STATA version 14 (StataCorp. 2015. Stata Statistical Software: Release 14. College Station, TX: StataCorp LP).

## Results

### Sample characteristics

In the 13-year program review period, of all the 245,257 patients ever enrolled on ART (as depicted in Table 1), females were slightly more (59.2%), majority (77.8%) of all clients resided in Nigeria’s northern states (Benue, FCT, Kano, Kaduna, Katsina, Nassarawa), were between 25 and 49 years (75.1%) in age. Most (98.9%) adults and paediatrics clients initiated a first line regimen at start of ART, half (50%) of enrollees had received ART for more than 25 months and 63.81% were on a bimonthly ARV refill schedule. Table 1 also shows that at study endpoint (30th June 2017), 97.4% (137,362/140,977) of the patients still on treatment were on first line regimen, which indicates that less than 3% switched from first to second line regimen, within this 13-year review. Among all ever enrolled on ART clients within the review period, 150,191 (61.2%) remained on treatment, 10,960 (4.5%) were transferred out to other facilities, 6926 (2.8%) died, 2139 (0.9%) self-terminated treatment and 75,041 (30.6%) had a LTFU event captured (see Table 1). Of the 75,041 LTFU clients, (67.2%) were female, resided mostly in the North (80.3%), were within ages of 25–49 years (73.6%) and received ART for greater than 25 months (33.7%) with 98.9% and 96.6% of them on first line regimen at start of ART and at time they were categorized as LTFU respectively. Of the 50,444 females recorded to have been LTFU, 1108 (2.2%) and 199 (0.4%) were pregnant or breastfeeding respectively.

### Comparing Patients who became LTFU and those retained in Care

In total, we evaluated and compared 75,041 patients who were recorded as LTFU and 150,191 who were retained in care (Tables 1, 2). Table 1 shows that 35.6% of Males were LTFU compared to 64.4% of them that are retained in care (p < 0.001), while 39.9% of the patients whose age group is 65 + as at 30th September, 2017 were LTFU as against their active counterparts (60.1%) in care (p < 0.001). Also 32.9% of pregnant females were LTFU in comparison to those retained (67.1%) in care (p < 0.001), while 34% of the patients from the Northern region (see Table 1 footnote) were LTFU compared to 66% of them retained in care (p < 0.001). Table 2 showed that a significantly lower proportion of adults on first line regimen at start of ART (33.4%) and at the end-point of the study (33.7%) are LTFU compared those retained in care at start of ART (66.6%) or at the end-point of the study (66.3%) at (p = 0.003, ART start and p < 0.001, end-point) respectively. Also, 49.4% of patients who received one-month ARV refills as seen in Table 2 were LTFU compared to 50.6% who were retained in care (p < 0.001) while 59.4% of those who were on ART for at most 6 months were LTFU compared to 40.6% of those retained in care (p < 0.001). Furthermore, 41.4% of those who had no viral load test done were LTFU compared to 58.6% that were retained in care (p < 0.001).

**Table 2:** Clinical factors and its association with LTFU

Maternal factors	LTFU		Total	p (< 0.05)
	No	Yes		
Regimen line at ART start				
Adult 1st line	140,977 (66.59)	70,719 (33.41)	211,696 (93.99)	*0.003*
Adult 2nd line	1530 (67.08)	751 (32.92)	2281 (1.01)	
Peadiatrics 1st line	7556 (68.24)	3516 (31.76)	11,071 (4.92)	
Peadiatrics 2nd line	128 (69.95)	55 (30.05)	183 (0.08)	
Regimen line at end-point of study				
Adult 1st line	137,362 (66.26)	69,937 (33.74)	207,299 (92.04)	< *0.001*
Adult 2nd line	6310 (73.75)	2246 (26.25)	8556 (3.80)	
Adult 3rd line/others	8 (80.00)	2 (20.00)	10 (0.00)	
Peadiatrics 1st Line	6265 (69.33)	2771 (30.67)	9036 (4.01)	
Peadiatrics 2nd Line	246 (74.32)	85 (25.68)	331 (0.15)	
Months of ARV refill				
1	26,314 (50.63)	25,662 (49.37)	51,976 (23.08)	< *0.001*
2	107,845 (73.53)	38,815 (26.47)	146,660 (65.12)	
≥ 3	16,032 (60.28)	10,554 (14.08)	26,596 (11.81)	
Duration on ART (month)				
≤ 6	22,697 (40.57)	33,249 (59.43)	55,946 (24.84)	< *0.001*
7–12 month	14,465 (67.08)	7098 (32.92)	21,563 (9.57)	
13–24	21,199 (69.20)	9434 (30.80)	30,633 (13.60)	
25+	91,823 (78.43)	25,258 (21.57)	117,081 (51.98)	
Current viral load (c/ml)				
Undetected	41,251 (92.65)	3271 (7.36)	44,552 (19.77)	< *0.001*
Detected	10,177 (82.55)	2152 (17.45)	12,329 (5.47)	
None	98,763 (58.65)	69,618 (41.35)	168,381 (74.76)	
Current viral load (c/ml)				
Suppressed	27,751 (96.44)	1025 (3.56)	28,776 (12.78)	< *0.001*
Unsuppressed	23,677 (84.33)	4398 (15.67)	28,075 (12.46)	
None	98,763 (58.65)	69,618 (41.35)	168,381 (74.76)	

p = p value which is in italics if significant at < 0.05

### Socio-demographic factors and their association with LTFU

As at end time of study (30th September 2017) as depicted in Table 1, a greater proportion of males (35.6%) compared to females (32.31%) were LTFU (p < 0.001). Also, the age group of 65 + years (39.9%) at endline of study accounted for the highest proportion of those LTFU followed by the age group of 50–64 years (34.51%) and 15–19 years (34.01%) respectively (p < 0.001). Pregnant females (32.9%) accounted for a higher proportion of those LTFU in comparison to those not pregnant (21.59%) or breastfeeding (11.56%) at a statistically significant value (p < 0.001). A higher proportion of patients from the northern region [see footnote of Table 1] (34%) accounted for LTFU compared to those from the southern part of the country (30.75%) and the difference is significant (p < 0.001). However, the proportion of LTFU in Kano state (28%) is less than that of Delta (36%) and Ogun (30%) states. There were also significant differences (p < 0.001) in LTFU across the states with the highest proportion of patients LTFU in Kaduna (45.97%), Katsina (36.40%) and Delta (36.10%) and the least proportion of LTFU patients in Osun (23.92%). Among the northern states, the North-central states of Benue, Nassarawa and FCT were responsible for the highest proportion of LTFU (34.13%) compared to the rest (33.43%) at p = 0.019.

### Clinical factors and their association with LTFU

Clinical factors and their association with LTFU are described in Table 2. During the implementation of the program, six first-line treatment regimens were prescribed: Zidovudine, lamivudine, and nevirapine or efavirenz (ZDV/3TC/NVP or EFV); stavudine, lamivudine, and nevirapine or efavirenz (d4T/3TC/NVP or EFV); and tenofovir, emtricitabine, and nevirapine or efavirenz (TDF/FTC/NVP or EFV). A greater proportion of adults on first line regimen (FLR) at start of ART (33.41%) were LTFU compared to those on second line regimen (SLR) at start of ART (32.92%) and this was similar for paediatrics (31.76% for FLR versus 30.05% for SLR) at p = 0.003. This FLR and SLR difference was also seen between adults (33.74% versus 26.25%) as well as paeditrics (30.67% versus 25.68%) on ART at the end-point of this study at p < 0.001. Patients who received one-month ARV refills (49.4%) accounted for more LTFU compared to those who received two-month (26.47%) or ≥ three-month refills (14.08%) at p < 0.001.

Table 2 also showed that a higher proportion of patients who were on ART for at most 6 months (59.4%) were LTFU compared to those on ART between 7 and 12 months (32.92%), 13–24 months (30.80%) or ≥ 25 months (21.57%) and this difference is statistically significant (p < 0.001).

Only about 25% of patients did a viral load test (the program between 2004 and 2013 was offering targeted VL monitoring) and a greater proportion of patients whose viral load was detectable (17.45%) were LTFU compared to those with undetectable viral load (7.36%) while a greater proportion of patients whose viral load was unsuppressed (15.67%) were LTFU compared to patients who were suppressed (3.56%) at p < 0.001.

### Predictors of LTFU

#### Univariate (Crude odds ratio) analysis

Univariate logistic regression analysis as shown in Table 3 showed that patients who were LTFU were more likely to be males (OR: 1.16, 95% CI 1.14–1.18, p < 0.001); non–pregnant females (OR: 3.76, 95% CI 3.24–4.35, p < 0.001); patients with un-suppressed viral load (OR: 2.67, 95% CI 2.52–2.83, p < 0.001) and those whose age were > 10 years (OR: 1.27, 95% CI 1.16–1.39, p < 0.001) with the strength of prediction increasing as the age increased to ≥ 65 years (OR: 1.81, 95% CI 1.67–1.97, p < 0.001). While patients who were less likely to be LTFU are those on two-monthly (OR: 0.37,95% CI 0.36–0.38. p < 0.001) or three-monthly ARV refills (OR: 0.68, 95% CI 0.66–0.70, p < 0.001); patients in Nigeria’s south (OR: 0.86, 95% CI 0.84–0.88, p < 0.001); patients whose viral load indication was targeted (OR: 0.57, 95% CI 0.53–0.62, p < 0.001); those whose facilities are located in the North-west region (OR: 0.97, 95% CI 0.94–0.99, p < 0.001); those on adult 2nd line regimen (OR: 0.70, 95% CI 0.67–0.73, p < 0.001), pediatric 1st line (OR: 0.87, 95% CI 0.83–0.91, p < 0.001) or pediatric 2nd line regimen (OR: 0.68, 95% CI 0.53–0.87, p = 0.002) and patients on ART for 7–12 months (OR: 0.33, 95% CI 0.32–0.35, p < 0.001) or 13–24 months (OR: 0.30, 95% CI 0.30–0.31, p < 0.001) or greater than 24 months (OR: 0.19, 95% CI 0.18–0.19, p < 0.001).

**Table 3:** Univariate and Multivariate analysis of predictors of LTFU

	Univariate (COR)		Multivariate (AOR) n = 156,124	
	OR [95% CI]	p	OR [95%CI]	p
Sex				
Female	1 [Ref]		1 [Ref]	
Male	1.16 [1.136–1.180]	< *0.001*	Omitted	
Female status				
Breastfeeding	1 [Ref]		1 [Ref]	
Pregnant	2.11 [1.792–2.477]	< *0.001*	1.83 [1.546–2.171]	< *0.001*
Not pregnant	3.76 [3.238–4.354]	< *0.001*	4.55 [3.893–5.307]	< *0.001*
Month of ARV refill				
1	1 [Ref]		1 [Ref]	
2	0.37 [0.361–0.377]	< *0.001*	0.59 [0.573–0.608]	< *0.001*
≥ 3	0.68 [0.656–0.696]	< *0.001*	1.32 [1.260–1.373]	< *0.001*
Region				
North^a^	1 [Ref]		1 [Ref]	
South^b^	0.86 [0.843–0.880]	< *0.001*	0.69 [0.671–0.712]	< *0.001*
Viral load indication				
Routine	1 [Ref]		1 [Ref]	
Targeted	0.57 [0.526–0.618]	< *0.001*	0.44 [0.393–0.487]	< *0.001*
Missing	6.06 [5.870–6.256]	< *0.001*	3.08 [1.247–7.616]	*0.015*
Regimen at ART start				
Adult 1st line	1 [Ref]		1 [Ref]	
Adult 2nd line	0.70 [0.666–0.734]	< *0.001*	1.23 [1.146–1.312]	< *0.001*
Paeds 1st line	0.87 [0.830–0.909]	< *0.001*	1.70 [1.376–2.112]	< *0.001*
Paeds 2nd line	0.68 [0.530–0.869]	= *0.002*	1.16 [0.707–1.888]	*0.564*
Viral result				
Suppressed	1 [Ref]		1 [Ref]	
Unsuppressed	2.67 [2.516–2.828]	< *0.001*	4.52 [4.109–4.961]	< *0.001*
Duration of ART (month)				
≤ 6	1 [Ref]		1 [Ref]	
7–12	0.33 [0.324–0.346]	< *0.001*	0.423 [0.407–0.443]	< *0.001*
13–24	0.30 [0.294–0.313]	< *0.001*	0.410 [0.394–0.426]	< *0.001*
25+	0.19 [0.184–0.192]	< *0.001*	0.246 [0.238–0.253]	< *0.001*
Age at LTFU (years)				
1–9	1 [Ref]		1 [Ref]	< *0.001*
10–14	1.27 [1.156–1.390]	< *0.001*	2.990 [2.557–3.496]	< *0.001*
15–19	1.40 [1.282–1.537]	< *0.001*	13.749 [10.458–18.077]	< *0.001*
20–24	1.23 [1.148–1.322]	< *0.001*	54.948 [40.407–74.721]	< *0.001*
25–49	1.36 [1.282–1.422]	< *0.001*	241.574 [176.541–330.565]	< *0.001*
50–64	1.44 [1.348–1.528]	< *0.001*	487.771 [354.269–671.582]	< *0.001*
65+	1.81 [1.666–1.967]	< *0.001*	1622.844 [1142.974–2304.185]	< *0.001*
Northern regions				
North central^c^	1 [Ref]		1 [Ref]	
North west^d^	0.969 [0.944–0.995]	= *0.019*	Omitted	

[Ref], reference; OR, odds ratio; CI, confidence interval; p, p value which is in italics if significant at < 0.05

^a^North also referred as Northern region: Benue, FCT, Kaduna, Kano, Katsina and Nassarawa

^b^South also referred as Southern region: Delta, Ondo, Ogun, Osun and Ekiti

^c^North central: Benue, FCT and Nassarawa

^d^North west: Kano, Kaduna and Katsina

#### Multivariate (adjusted odds ratio) analysis

The multivariate logistic regression model (see Table 3) was significant and able to account for 93% (Pseudo R^2^ = 0.1733) of the variables that predicted LTFU among 156,124 patients. Non-pregnant female patients on ART were about 5 times (aOR: 4.55, 95% CI 3.89–5.31, p < 0.001) more likely to be LTFU compared to their breastfeeding counterparts, while patients on ≥ 3-monthly ARV refills were more likely (aOR: 1.32, 95% CI 1.26–1.37, p < 0.001) to be LTFU compared to those on one-month ARV refills, while those on two-month refill were less likely (aOR: 0.59, 95% CI 0.57–0.61, p < 0.001) to be LTFU compared to those on one-month ARV refills. Patients with un-suppressed viral loads on ART have about 3 times (aOR: 4.52, 95% CI 4.11–4.96, p < 0.001) increased odds of being LTFU compared to their suppressed counterparts. Patients on adult 2nd line regimen (aOR: 1.23 95% CI 1.15–1.31, p < 0.001) or paediatric 1st line regimen (aOR: 1.70 95% CI 1.38–2.11, p < 0.001) were significantly more likely to be LTFU compared to counterparts on adult 1st line regimen. The likelihood of being LTFU in comparison to patients aged 1–9 years, increased with increasing age from 10 to 14 years (aOR: 2.99 95% CI 2.56–3.50, p < 0.001) to its highest at ≥ 65 years (aOR: 1622.84 95% CI 1142.97–2304.19, p < 0.001). Meanwhile as the duration on ART of patients increased from 7 to 12 months (aOR: 0.42, 95% CI 0.41–0.44, p < 0.001) to 13–24 months (OR: 0.41 95% CI 0.39–0.43, p < 0.001) and ≥ 25 months (aOR: 0.25 95% CI 0.24–0.25, p < 0.001) the less likely were patients LTFU in comparison to patients on ART for less than 6 months. Also, patients who are in southern part of Nigeria (aOR: 0.69, 95% CI 0.67–0.71, p < 0.001) compared to the North or whose indication for viral load test was targeted (aOR: 0.44, 95% CI 0.39–0.49, p < 0.001) rather than routine were significantly less likely to be LTFU.

## Discussion

The proportion of patients LTFU in this study is similar to the findings of studies conducted in Uganda and Nigeria where the cumulative incidence of LTFU at 2 years was 30% [5] and 37.7% at 3 years [17] respectively. This is in contrast to the 7.3% LTFU reported in a study in Togo [18], 14.7% LTFU reported in a retrospective study in Kwazulu Natal, South Africa [19] or 19% prevalence of LTFU in a study involving 15,000 patients in 5 West African countries [20], while a study in Guinea Bissau reported a LTFU prevalence of 57.61% after 7 years [21].

This variation in prevalence rates of LTFU might be due to heterogeneity of the population groups studied and the unstandardized definition of LTFU [22], however it shows that high LTFU rates is a consistent challenge affecting ART programs especially in sub-Saharan Africa [2, 23]. Designing effective referral systems and strengthening them for tracking LTFU patients and returning them back to care is essential in preventing programmatic HIV cascade loss [2, 22].

Although more females were in this study, men were more likely to be LTFU in concurrence with similar studies [14, 16, 20, 21, 24–28] where male gender was identified as an independent predictor of LTFU in the HIV/AIDS program. In contrast to our findings, a study in Togo reported that females were more likely to be LTFU compared to males [18]. It was suggested in a study that men are predisposed to LTFU because they present late for care compared to women and may be at increased risk of severe illness and death [27]. Increased risk of LTFU amongst men indicates need for specific gender based intervention to increase retention [16]. Furthermore, the impact of antenatal care entry and in extension prevention of mother to child transmission programs on eventual retention of participants requires study.

Increasing PLHIV age was associated with LTFU, with the elderly age group (> 65 years) at higher risk of LTFU than the pediatric age group (1–9 years);this aligns with findings from a previous study [24, 25] but is incongruent with results from a case–control study done in Ethiopia’s Oromia region, which showed that patients aged 15–24 years were about 19 times more likely to be LTFU on ART treatment compared to those patients aged above 55 years [29]. Nigeria’s vital registration systems (birth and death registration) is shaky and many deaths are likely captured as LTFU, among the elderly (> 65 years) this could be a factor especially in relation to all-cause vs AIDS-related mortality. Further research of age specific LTFU rate especially amongst adolescents living with HIV who are transiting into adult HIV care should also be explored.

LTFU in our study was more common in the first 6 months of ART initiation as reported in previous studies [22, 24]. New ART initiates may need to navigate psychosocial barriers to remain in care and should be targeted for additional comprehensive adherence counselling [30]. An inverse relationship between LTFU risk and increasing monthly duration of ARV refills was noted with more patients LTFU that are on a monthly ARV refill. Clinicians can adjust ARV refill based on client staging. Those on monthly revisits were likely of lower immunological staging, which also predisposes them to high LTFU risk. Providing longer duration of refills for stable patients can reduce LTFU attributable to high transportation or away from occupation costs of seeking care [22].

Our study showed geographical variation in the risk of LTFU with patients residing in the North with increased risk (except in Kano) compared to the south and patients from the North-Central region more likely to be LTFU compared to the North-West region. This geographical variation has been reported in a previous Nigerian study although with a different pattern [15, 16]. The high HIV- prevalence settings of Nigeria’s North Central relative to its health workforce availability might be a contributing factor.

Long duration of observation of the cohorts on ART and the large number of the patients analyzed improves generalizability of our findings. However, this study is limited by the unavailability of some demographic, clinical and laboratory information that are important variables that could predict LTFU including but not limited to CD4 count, weight, educational status and adherence to ART.

## Conclusion

This study demonstrates that LTFU is still a challenge in the HIV program in Nigeria. Male gender, 2nd line regimen, increasing age group interval from 9 years, un-suppressed viral load or targeted viral load test indication, residence in the north especially North Central region, increasing duration of ARV refills and ART initiation of less than 6 months are all significant predictors of LTFU in Nigeria. Differentiated care approaches are advocated.

## Data Availability

The datasets generated and/or analysed during the current study are not publicly available due to institutional policy but are available from the corresponding author on reasonable request.

## Abbreviations

AIDS: acquired immuno-deficiency syndrome
ART: anti-retroviral therapy
ARV: anti-retroviral
cART: combined Anti-retroviral therapy
CD4: cell differentiated 4
CI: confidence Interval
FLR: first line regimen
HIV: human immune-deficiency virus
IHVN: Institute of Human Virology Nigeria
LTFU: loss to follow-up
NHREC: National Health Research Ethics Committee
OR: odds ratio
PEPFAR: President’s Emergency Plan for AIDS Relief
PLHIV: people living with Human Immunodeficiency Virus
p: p-value
SLR: second line regimen
STATA: StataCorp. 2015. Statistical Software: Release 14. College Station TX
